# Novel reinforcement learning technique based parameter estimation for proton exchange membrane fuel cell model

**DOI:** 10.1038/s41598-024-78001-5

**Published:** 2024-11-11

**Authors:** Nermin M. Salem, Mohamed A. M. Shaheen, Hany M. Hasanien

**Affiliations:** 1 Electrical Power and Machines Department, Faculty of Engineering, Ain Shams University, Cairo, 11517 Egypt; 2 Faculty of Engineering and Technology, Future University in Egypt, Cairo, 11835 Egypt

**Keywords:** Clean energy, PEMFC, Reinforcement learning, Fuel cells, Electrical and electronic engineering

## Abstract

Proton Exchange Membrane Fuel Cells (PEMFCs) offer a clean and sustainable alternative to traditional engines. PEMFCs play a vital role in progressing hydrogen-based energy solutions. Accurate modeling of PEMFC performance is essential for enhancing their efficiency. This paper introduces a novel reinforcement learning (RL) approach for estimating PEMFC parameters, addressing the challenges of the complex and nonlinear dynamics of the PEMFCs. The proposed RL method minimizes the sum of squared errors between measured and simulated voltages and provides an adaptive and self-improving RL-based Estimation that learns continuously from system feedback. The RL-based approach demonstrates superior accuracy and performance compared with traditional metaheuristic techniques. It has been validated through theoretical and experimental comparisons and tested on commercial PEMFCs, including the Temasek 1 kW, the 6 kW Nedstack PS6, and the Horizon H-12 12 W. The dataset used in this study comes from experimental data. This research contributes to the precise modeling of PEMFCs, improving their efficiency, and developing wider adoption of PEMFCs in sustainable energy solutions.

**Supplementary Information:**

The online version contains supplementary material available at 10.1038/s41598-024-78001-5.

## Introduction 

The environmental concerns about fossil fuels have accelerated the search for clean and sustainable energy sources^1^. Fuel cells are one of the most promising options since they can directly transform chemical energy into electrical energy through electrochemical processes^2^. They are efficient and can generate power with low gas emissions^3^. Several fuel cell types have been used, such as MCFCs^4^, SOFCs^5^, and PEMFCs^6^. Although SOFCs and MCFCs function at higher temperatures^7^, PEMFCs are well-known for their mobility^8^, making them suitable for energy supply in residential^9^, commercial^10^, and industrial applications^11^.

PEMFC is supposed to play a vital role in a cleaner and more sustainable future^12^. This is because of its advantages, including low emissions and high efficiency^13^. PEMFC has the potential to contribute to solving the problems related to pollution and dependence on fossil fuels^14,15^. Research studies focus on various applications for PEMFCs, such as microgrids^16,17^. Accurate models are necessary to analyze PEMFC performance and validate software simulations using experimental data.

Numerous approaches have been recommended for modeling PEMFCs. These approaches include analytical^18^, empirical^19^, and theoretical methods^20,21^. The Analytical methods use mathematical equations and physical principles to describe the behavior of PEMFC. The empirical methods depend on data to get relationships between input and output variables. The Theoretical methods develop mathematical models that represent the physical processes of a PEMFC. Theoretical models, both conventional and metaheuristic, are commonly used in research on PEMFC parameters extraction. The methodology introduced in^22^ uses a hybrid analytical with a Computational Fluid Dynamics model to optimize the thermos fluid performance of fuel cells. Reference^23^ uses a semi-empirical model to analyze voltage performance in PEMFC stacks, considering friction losses. Experimental data also validate the study of that research. In^24^, the PEMFC modeling is based on a semi-empirical approach where electrical, thermal, and degradation models simulate PEMFC performance. The approach of such reference is designed to optimize efficiency and lifetime for naval applications. With notable advancements in computing, various meta-heuristic algorithms can be used to solve this problem such as chaos game optimization algorithm^25^, Walrus optimization algorithm^26^, Coot Bird Algorithm^27^, Sunflower Optimization Algorithm^28^, and Transient Search Algorithm^29,30^. The WOA approach is validated in^31^ by comparing the model estimated results with experimental data from various PEMFC systems under different conditions.

Recently, there has been interest in applying RL methods to PEMFC modeling. RL offers a promising alternative to traditional methods, as it can learn from data and adapt to changing conditions^32,33^. RL is a subset of machine learning that performs superiorly in environments where direct solutions are hard to compute due to complex, nonlinear relationships^34,35^. RL and other data-driven methods have demonstrated significant potential in computing prediction methodology for various applications^36,37^, including Fuel Cell Performance Prediction, providing a promising alternative to the traditional approach. Instead of relying on predefined rules, an RL agent learns through interaction with the system and receives feedback (rewards or penalties) based on its actions^38^. Despite its promising performance, RL remains underutilized in the energy sector, presenting a clear need for further exploration and research^39^. However, recent progress in RL, especially in gradient-based methods like actor-critic algorithms, has significantly improved the ability to learn effective performance prediction model for complex systems.

RL can be classified into several categories based on how the learning process is defined and the types of environments the agent interacts with. For the complex nature of PEMFC optimization, Actor-Critic methods are particularly well-suited for tasks with continuous action spaces, as they can directly learn policies that output continuous actions^40^; it involves tuning continuous nonlinear parameters. PEMFCs are governed by multiple nonlinear parameters, which affect the system’s behaviour in intricate ways. This makes them an ideal candidate for RL approaches that balance value-based and policy-based learning.

Actor-critic algorithms are a class of reinforcement learning methods that combine two key networks: the actor $\:{\pi\:}_{\theta\:}\left(s,a\right),$ which maps states s to a probability distribution over actions 𝑎, which determines the best action to take in each state, and the critic, value function V(s), which evaluates the action by estimating the value function. The actor updates its policy based on feedback from the critic, while the critic continuously improves its value estimations based on the rewards received. This actor-critic acts as a two-player game that allows for more stable and efficient learning than standalone policy-based or value-based methods. Actor-critic methods are particularly effective and well-suited in environments with complex, continuous state-action spaces in which decisions involve multiple variables and nonlinear dynamics.

Indeed, the fuel cell optimization problem is considered challenging and focuses on unlocking new opportunities for applying RL. In^41^ The authors introduced an RL model that combines the Proximal Policy Optimization (PPO) algorithm with the REINFORCE update rule for optimizing both the design and prediction of the robotic environment, outperforming other methodologies used in^42^ and^43^. In^44^, the authors adopted a combined policy gradient with a model for optimizing photovoltaic and battery.

In this work, a PPO agent, a type of actor-critic RL method, is employed as an advanced reinforcement learning tool to optimize PEMFCs by minimizing the squared error between measured and simulated terminal voltage, which is governed by seven nonlinear parameters. PPO, an on-policy gradient method, accomplishes a balance between exploring new actions and exploiting known information by constraining the update step size based on the most recent experiences collected during training, which avoids divergence issues while training and guarantees adaptability to change environments^45^. The PPO agent iteratively interacts with the PEMFC system, adjusting the nonlinear parameters based on the reward feedback related to the voltage error reduction. By gradually refining its policy through this interaction, the agent learns an optimal prediction strategy to align the simulated voltage closely with the actual measurements.

The proposed PPO model is also a model-free reinforcement learning approach as it does not require a predefined mathematical model of the environment^46^. It learns directly from environmental interactions, making it versatile and applicable to PEMFCs without an explicit system model.

The proposed on-policy, model-free PPO’s advantage lies in its ability to handle complex, continuous prediction problems, making it well-suited for optimizing the intricate dynamics of PEMFCs. This approach offers a robust solution for enhancing fuel cell performance, offering improvements over traditional performance prediction models.

The main contribution of this paper is to present a novel reinforcement learning-based approach for optimizing PEMFC nonlinear parameters and improving the accuracy of PEMFC model under different operating conditions of temperature and pressure for different types of PEMFCs. The remaining sections of the paper are presented as follows. Section 2 provides the mathematical representation of the PEMFC. Section 3 provides the objective function and the constraints. Section 4 provides the methodology. Section 5 includes a discussion of the simulation results. Section 5 presents conclusions and future work for this study.

## Mathematical Modelling of PEMFC and objective function formulation

A detailed PEMFC model can be found in^47^. The PEMFC voltage is computed as shown in Eq. (1). In this Equation, $\:{N}_{cells}$ is the number of series cells. The total of the voltages across all the individual cells is the voltage of the stack.

1 $$\:{\varvec{V}}_{\varvec{s}\varvec{t}\varvec{a}\varvec{c}\varvec{k}}={\varvec{N}}_{\varvec{c}\varvec{e}\varvec{l}\varvec{l}\varvec{s}}.\left({\varvec{E}}_{\varvec{n}\varvec{e}\varvec{r}\varvec{n}\varvec{s}\varvec{t}}-{\varvec{v}}_{\varvec{a}\varvec{c}\varvec{t}}-{\varvec{v}}_{\varvec{\varOmega\:}}-{\varvec{v}}_{\varvec{c}\varvec{o}\varvec{n}\varvec{c}}\right)$$

Here, $\:{E}_{nernst}$ refers to the Nernst potential. It computes the PEMFC OC voltage, which is calculated as shown in Eq. (2). $\:{v}_{act}$ denotes the activation overpotential. $\:{v}_{act}$ denotes the V_loss_ caused by the resistance. $\:{v}_{conc}$ denotes the concentration overpotential. Mathematically, $\:{\text{v}}_{\text{a}\text{c}\text{t}},\:{\text{v}}_{{\Omega\:}},$ and $\:\:{\text{v}}_{\text{c}\text{o}\text{n}\text{c}}$ are computed as in Eqs. (3)-(5).

2 $$\:{\varvec{E}}_{\varvec{N}\varvec{e}\varvec{r}\varvec{n}\varvec{s}\varvec{t}}=1.229-0.85\times\:{10}^{-3}\left({\varvec{T}}_{\varvec{f}\varvec{c}}-298.15\right)+4.3085\times\:{10}^{-5}{\varvec{T}}_{\varvec{f}\varvec{c}}\varvec{ln}\left({\varvec{P}}_{{\varvec{H}}_{2}}\sqrt{{\varvec{P}}_{{\varvec{O}}_{2}}}\right)$$

where, $\:{T}_{{fc}}$ is the temperature in Kelvin. $\:{P}_{{H}_{2}}$ and $\:{P}_{{O}_{2}}$ are the partial pressures of hydrogen and oxygen.

3 $$\:{\varvec{v}}_{\varvec{a}\varvec{c}\varvec{t}}=-\left[{\varvec{\xi\:}}_{1}+{\varvec{\xi\:}}_{2}{\varvec{T}}_{\varvec{f}\varvec{c}}+{\varvec{\xi\:}}_{3}{\varvec{T}}_{\varvec{f}\varvec{c}}\varvec{ln}\left({\varvec{C}}_{{\varvec{O}}_{2}}\right)+{\varvec{\xi\:}}_{4}{\varvec{T}}_{\varvec{f}\varvec{c}}\varvec{ln}\left({\varvec{I}}_{\varvec{f}\varvec{c}}\right)\right]$$

where $$\:{\varvec{C}}_{{\varvec{O}}_{2}}=\frac{{\varvec{P}}_{{\varvec{O}}_{2}}}{5.08.{10}^{6}}.\:\varvec{e}\varvec{x}\varvec{p}(498/{\varvec{T}}_{\varvec{f}\varvec{c}})$$

4 $$\:{\varvec{v}}_{\varvec{\varOmega\:}}={\varvec{I}}_{\varvec{f}\varvec{c}}\left({\varvec{R}}_{\varvec{m}}+{\varvec{R}}_{\varvec{c}}\right);\:{\varvec{R}}_{\varvec{m}}=\frac{{\varvec{\rho\:}}_{\varvec{m}}\varvec{l}}{{\varvec{M}}_{\varvec{A}}}$$

where $$\:{\varvec{\rho\:}}_{\varvec{m}}=\frac{181.6\left[1+0.03\left(\frac{{\varvec{I}}_{\varvec{f}\varvec{c}}}{{\varvec{M}}_{\varvec{A}}}\right)+0.062{\left(\frac{{\varvec{I}}_{\varvec{f}\varvec{c}}}{303}\right)}^{2}{\left(\frac{{\varvec{I}}_{\varvec{f}\varvec{c}}}{{\varvec{M}}_{\varvec{A}}}\right)}^{2.5}\right]}{\left[\varvec{\lambda\:}-0.634-3\left(\frac{{\varvec{I}}_{\varvec{f}\varvec{c}}}{{\varvec{M}}_{\varvec{A}}}\right)\right].\varvec{e}\varvec{x}\varvec{p}\left[4.18\left(\frac{{\varvec{T}}_{\varvec{f}\varvec{c}}-303}{{\varvec{T}}_{\varvec{f}\varvec{c}}}\right)\right]}$$

5 $$\:{\varvec{v}}_{\varvec{c}\varvec{o}\varvec{n}\varvec{c}}=-\varvec{\beta\:}.\varvec{ln}\left(1-\frac{\varvec{J}}{{\varvec{J}}_{\varvec{m}\varvec{a}\varvec{x}}}\right)$$

Here, $\:{\xi\:}_{1}$ to $\:{\xi\:}_{4}$ as well as $\:\beta\:$ represent empirical coefficients. $\:{C}_{{O}_{2}}$ denotes the oxygen concentration. $\:{I}_{fc}$ denotes the current. $\:{R}_{m}$ and $\:{R}_{c}$ denote the membrane and contact resistances. $\:{\rho\:}_{m}$ denotes the membrane resistivity. $\:l$ denotes the thickness. $\:{M}_{A}$ denotes the surface area. $\:J$ denotes the current density. $\:\lambda\:$ also represents a design variable. The defined design variables in this study are ($\:{\xi\:}_{1}$, $\:{\xi\:}_{2}$, $\:{\xi\:}_{3}$, $\:{\xi\:}_{4}$, λ, $\:{R}_{c}$, and $\:\beta\:$). The PEMFC is represented by seven nonlinear parameters directly influencing the fuel cell output voltage. The goal of the optimization task is to minimize the SSE between the measured terminal voltage and the simulated voltage, modeled by the equations governing fuel cell behavior^48^. Equation (6) provides the goal function’s mathematical formulation.

6 $$\:\varvec{S}\varvec{S}\varvec{E}=\sum\:_{\varvec{m}=1}^{{\varvec{N}}_{\varvec{s}\varvec{a}\varvec{m}\varvec{p}\varvec{l}\varvec{e}\varvec{s}}}{\left[{\varvec{V}}_{\varvec{F}\varvec{C},\varvec{e}\varvec{x}\varvec{p}}\left(\varvec{m}\right)-{\varvec{V}}_{\varvec{F}\varvec{C},\varvec{e}\varvec{s}\varvec{t}}\left(\varvec{m}\right)\right]}^{2}$$

where, $\:{\text{N}}_{\text{s}\text{a}\text{m}\text{p}\text{l}\text{e}\text{s}}$ is the number of experimental readings. $\:{\text{V}}_{\text{F}\text{C},\text{e}\text{x}\text{p}}$ is the experimental voltage. $\:{\text{V}}_{\text{F}\text{C},\text{e}\text{s}\text{t}}$ denotes the estimated voltage. Limits, expressed as inequality constraints, apply to the design variables. To optimize the goal, the suggested RL method was conducted using Google Colab notebooks.

## Reinforcement learning for PEMFC Parameter Estimation

This section outlines the proposed RL approach for system prediction and then details the customization made to enable learning system designs. The methodology involves designing a custom environment in accordance with the PEMFC model and applying the PPO algorithm to optimize seven nonlinear parameters iteratively to minimize the sum of squared errors (SSE) between measured and estimated fuel cell voltages. Three different cells were tested, and our proposed RL model outperforms other traditional methods.

The problem is simulated as a reinforcement learning task, where the agent’s objective is to minimize the SSE between the actual and simulated voltage by adjusting the seven nonlinear parameters.

### Environment design

A custom reinforcement learning environment was developed using the gymnasium interface, specifically tailored to the PEMFC system. The software used in this study was created in Python, and the development was done in a GPU-based Google Colab notebook. We relied on the Stable Baselines3 library to build the reinforcement learning agent utilizing the Proximal Policy Optimization (PPO) algorithm. The agent’s interactions occurred within a custom environment set up and managed using Gymnasium. As the agent made decisions and received feedback, NumPy handled all the necessary numerical calculations and data processing. Finally, to better understand the results and monitor the learning process, Matplotlib was used to generate visual plots.

The environment defines the state space, action space, and reward structure as follows:

- State Space s: The state space consists of the seven nonlinear parameters influencing the fuel cell’s voltage output. Based on empirical data, these parameters are initialized randomly within predefined bounds.
- Action Space a: The action space is continuous and represented by a seven-dimensional vector, where each action corresponds to an adjustment of one of the nonlinear parameters within a normalized range of [-1, 1].
- Reward Function R: The reward is calculated as the negative of the SSE between the measured and simulated terminal voltages. When the SSE falls below a threshold value, an additional reward bonus of 1000 is provided, encouraging the agent to achieve an accurate simulation.
- Termination Criteria: The episode terminates when the SSE falls below a threshold value, indicating sufficient optimization of the parameters or when a predefined maximum number of steps is reached.

The threshold value is defined based on the type of cell tested; it is the minimum SSE achieved for each cell based on the literature review. This threshold value encourages the agent to achieve approximately equal voltage values for the cells tested.

### PPO agent training

The PPO algorithm from the `stable-baselines3` library was selected for training the agent due to its robustness in handling continuous action spaces and efficiency in large-scale optimization tasks. The agent’s policy was modeled using a multi-layer perceptron (MLP) network. Two networks are created, one for the actor and one for the critic. The actor-network is responsible for outputting a probability distribution over actions. At the same time, the critic network estimates the current state’s value, which helps evaluate how good the chosen action was, providing feedback to the actor. Both MLP networks consist of fully connected layers with nonlinear activation functions, and a Rectified Linear Unit (ReLU) is applied after each fully connected layer to introduce nonlinearity. This nonlinearity is crucial for the network to learn complex representations and relationships within the data.

The PPO agent was trained using the following hyperparameters:

- Learning Rate: 0.0001.
- n_steps: 2048 steps per iteration.
- Batch Size: 64.
- n_epochs: 10.
- Gamma (discount factor): 0.99.
- Clip Range: 0.2.
- Entropy Coefficient: 0.01.

These hyperparameters were selected to balance exploration and exploitation while ensuring stable convergence during training.

### Training process

The training process involved initializing the environment with random values of the nonlinear parameters and then allowing the PPO agent to interact with the environment by adjusting the parameters iteratively. At each step, the agent modified the parameters and received feedback as a reward based on the calculated SSE. The agent aimed to minimize the SSE by learning an optimal policy for parameter adjustment. The training was conducted over total_timesteps = 10,000, corresponding to ten epochs, each consisting of an entire episode during which the agent was allowed to interact with the environment until termination. After each iteration, the best-performing set of parameters was recorded based on the lowest SSE achieved. The training lasted for 5 min on a GPU (T4) -based Google Colab Notebook.

Algorithm 1 outlines the optimization process. It begins with initializing the design distribution, facilitating an extensive exploration of designs. Throughout the training process, the framework fine-tunes the policy parameters θ and design parameters ϕ to gradually phase out less effective designs. This enables the policy to specialize and concentrate on a narrowing set of promising designs. Consequently, the variance within the design distribution decreases, steering the system towards converging on an optimal design. The policy is updated using the clipped surrogate objective: $L_{clip}=\min(\mathrm r(\mathrm\theta)\ast{\mathrm A}_{\mathrm t},\mathrm{clip}(\mathrm r(\mathrm\theta),1-\mathrm\epsilon,1+\mathrm\epsilon)\ast{\mathrm A}_{\mathrm t}$, where $\mathrm r(\mathrm\theta)$ = $\frac{{\mathrm\pi}_\theta({\mathrm a}_{\mathrm t}/{\mathrm s}_{\mathrm t})}{{\mathrm\pi}_{\theta\_old}({\mathrm a}_{\mathrm t}/{\mathrm s}_{\mathrm t})}$ is the ratio between the new and old policies and $A_t$ is the estimated advantage. The value function is updated by minimizing the squared error between the predicted value and the actual return $L_{value}(\theta)=(R_t-V{(s_t))}^2$, where is the discounted return. The entropy is used to encourage exploration $L_{entropy}(\theta)=-\mathbb{E}\lbrack Entropy({\mathrm\pi}_\theta)\rbrack$, The entropy coefficient ​ determines how much weight is given to exploration. The total loss function combines the clipped policy objective, value function loss, and entropy bonus ​$L_{total}=-L_{clip}+c_1L_{value}-c_2L_{entropy}$, where $c_1$ and $c_2$ are scaling factors that balance the different components.

**Table:** 

Algorithm 1
Initialize policy (actor) network $\:{\pi\:}_{\theta\:}(s,a)$ and value (critic) network $\:{V}_{\theta\:}\left(s\right)$ **Initialize environment-specific parameters for PEMFC cell.** **Set** PPO hyperparameters (learning rate, batch size, clip range, etc.)
**repeat** • **Sample experience tuples** {$\:{s}_{t},\:{a}_{t},\:{r}_{t},\:{s}_{t+1}$}over n_steps from the environment using $\:{\pi\:}_{\theta\:}$.​ • **Compute advantages**$\:{A}_{t}\:$and **discounted rewards**$\:{R}_{t}$: $\:{A}_{t}\:=\:{R}_{t}-V\left({s}_{t}\right)$ $\:{R}_{t}={r}_{t}+\:\gamma\:*V({s}_{t}+1)\:$ • **Update value network**$\:{V}_{\theta\:}$ by minimizing the loss: L(V) = $\:\frac{1}{N}\sum\:{({R}_{t}-\:V({s}_{t}\left)\right)}^{2}$ • **Update policy network**$\:{\pi\:}_{\theta\:}$ using PPO objective with gradient descent: $\:\text{C}\text{o}\text{m}\text{p}\text{u}\text{t}\text{e}\:\text{r}\text{a}\text{t}\text{i}\text{o}\:r\left(\theta\:\right)\:=\:\frac{{\pi\:}_{\theta\:}\:\left({a}_{t}/{s}_{t}\right)}{{\pi\:}_{\theta\:\_old}\:\left({a}_{t}/{s}_{t}\right)}$ $\:{L}_{clip}=\text{m}\text{i}\text{n}(r\left(\theta\:\right)*{A}_{t},\:clip\left(r\left(\theta\:\right),\:1-\mathrm\epsilon,\:1+\mathrm\epsilon\right)*{A}_{t}$ **Final loss for policy update** $\:{L}_{total}=\:-{L}_{clip}+{c}_{1}*{L}_{value}-\:{c}_{2}*Entropy\left({\pi\:}_{\theta\:}\right)$ • **Backpropagate and update** policy and value networks.
Until the End of training

## Results, discussion, and insights

The performance of the PPO agent was evaluated by tracking the progression of SSE over time and recording the best set of parameters that minimized the voltage error. In the testing phase of our RL model, we performed a comprehensive evaluation to assess the performance of the trained agent. The raw data collected during testing were used in the visualization phase to gain insights into the model’s behavioral pattern and its effectiveness in minimizing the error metrics. The analysis involved plotting the values of SSE, reward, and Zeta parameters for each iteration. All agents were trained for total_timesteps = 10,000, corresponding to ten epochs.

The partial pressure of both oxygen and hydrogen was maintained at 0.5 atm for all experiments. Additionally, the operating temperature was consistently set at 50 °C for all cases.

### Case study 1: Temasek 1 kW PEMFC

**Figure 1:**
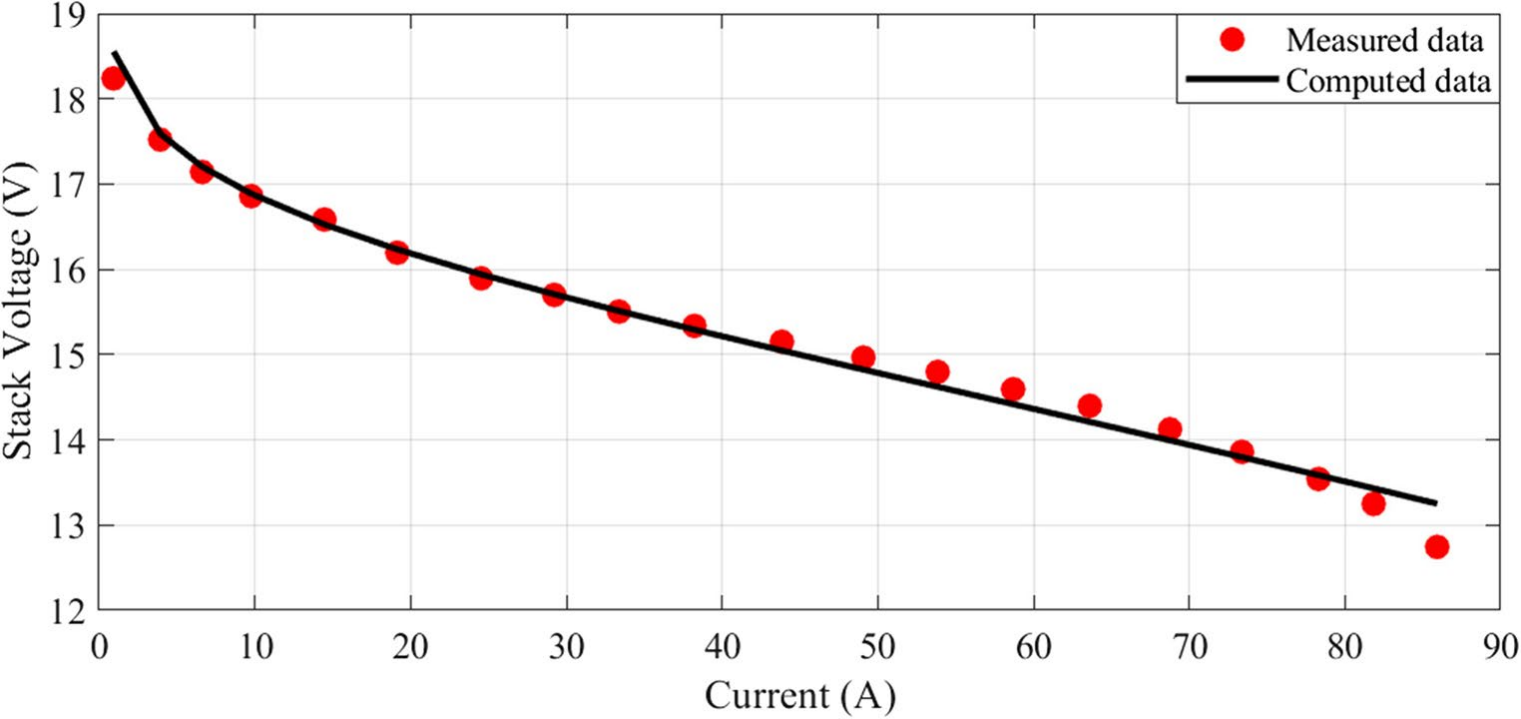
I-V Curves for Case 1.

**Figure 2:**
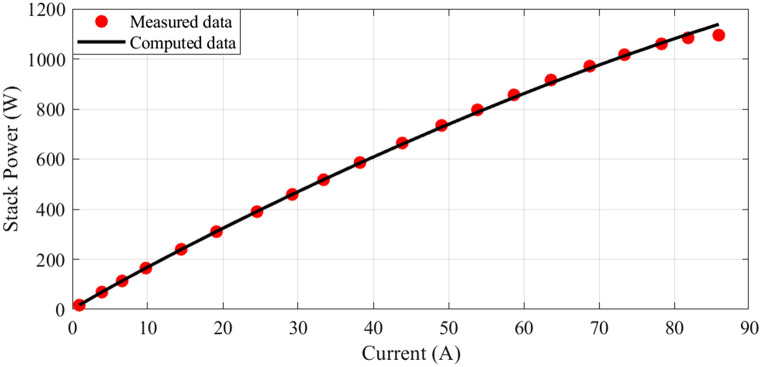
I-P Curves for Case 1.

In this scenario, there are 20 series-connected cells with a total active area of 150 cm². The maximum current density is 1.5 A/cm². The I-V characteristics are depicted in Fig. 1, and a comparison between the calculated and measured voltages is presented. Additionally, Fig. 2 illustrates the relationship between current and power.

Table 1 compares optimization techniques with the RL approach. It displays the best possible candidate solutions for a range of design variables and provides the best results for reducing the SSE between the observed and estimated terminal voltages achieved by each approach.

**Table 1:** Design variables for case 1.

Parameter	RL	EWO^49^	KOA^49^	MPA^49^	HHO^49^
$\:{\xi\:}_{1}$	-1.0789201	-0.881369628	-0.8731	-0.9777	-0.8532
$\:{\xi\:}_{2}$	0.003271523	0.002988173	2.7642	3.424	0.002329774
$\:{\xi\:}_{3}$	5.19432E-05	7.44626E-05	6.13E-05	4.97E-05	3.60E-05
$\:{\xi\:}_{4}$	-9.54E-05	-0.0000954	-9.5	-23.6873	-0.0000954
λ	10	13	13	10	13
$\:{R}_{c}$	1E-04	0.0001	0.0001	0.0001	0.0008
β	0.13017318	0.163327329	0.1619	0.0225	0.0136
SSE	0.559348311	0.578753177	0.590467	0.7559	0.825511853

**Figure 3:**
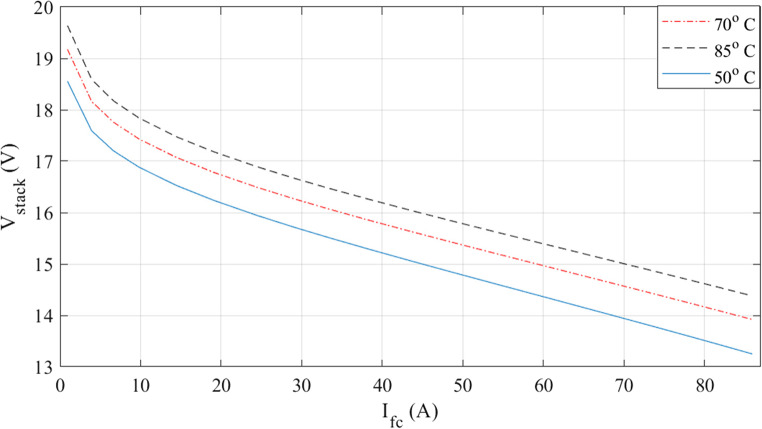
I-V curves at various temperatures for Case 1.

**Figure 4:**
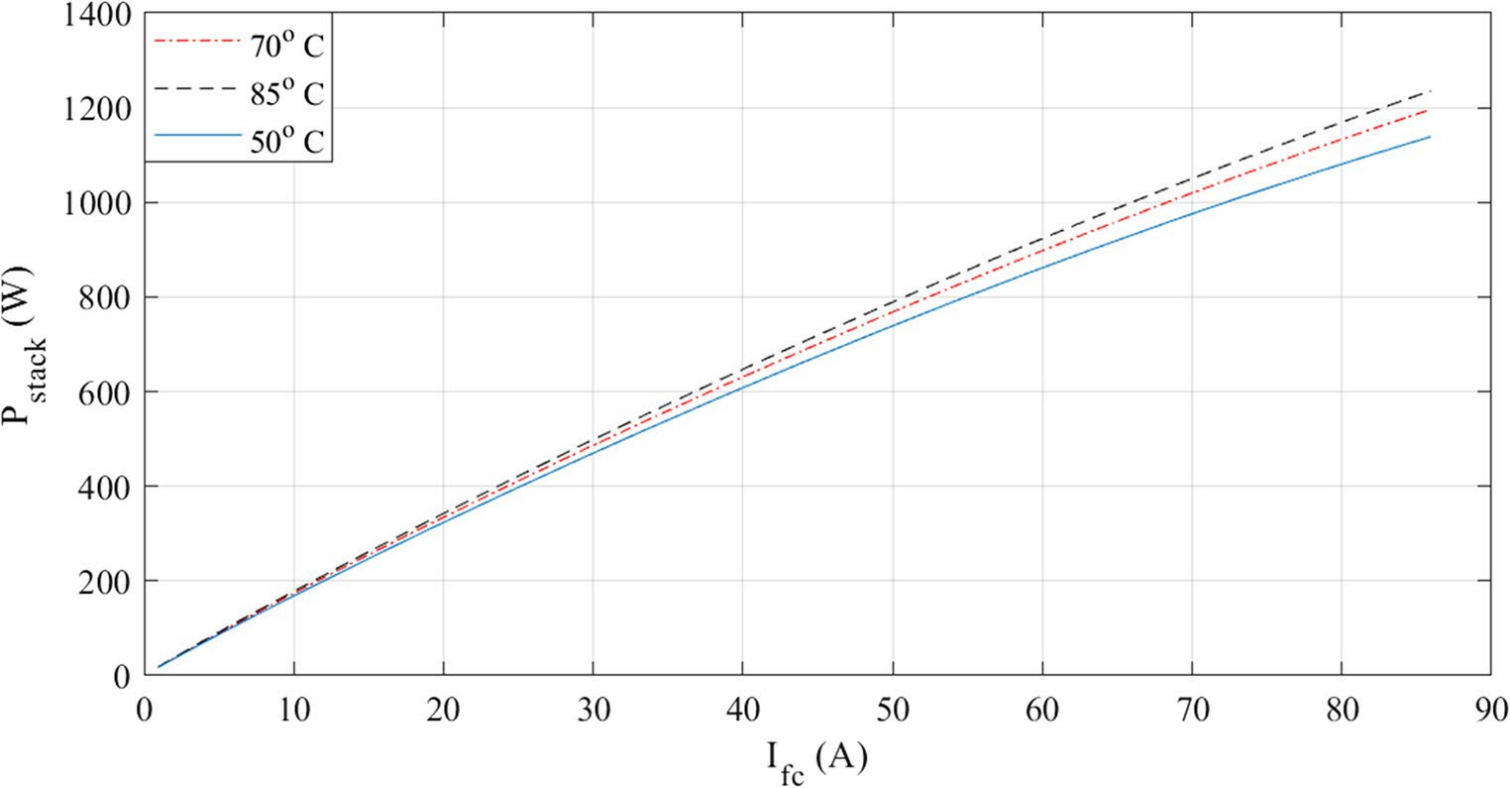
I-P curves at various temperatures for Case 1.

The characteristics at different temperatures are examined in the next two figures. I-V waveforms at 50, 70, and 85 °C are shown in Fig. 3. The plot shows that the voltage for a given current increase when the temperature rises. Additionally, Fig. 4 shows how the I-P curves compare at different temperatures. The power curves demonstrate how a little temperature variation affects the output power.

**Figure 5:**
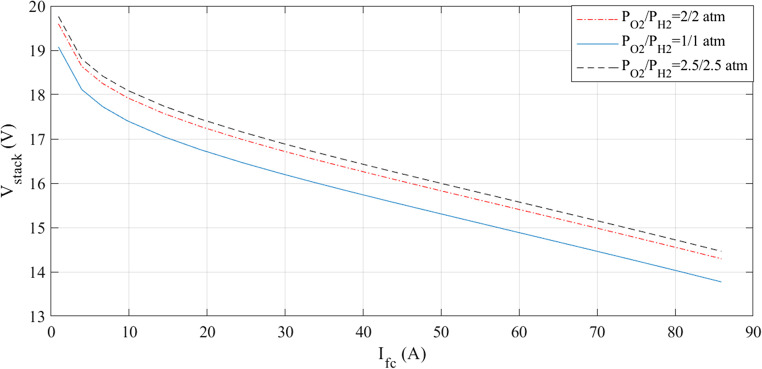
I-V curves at various pressures for Case 1.

**Figure 6:**
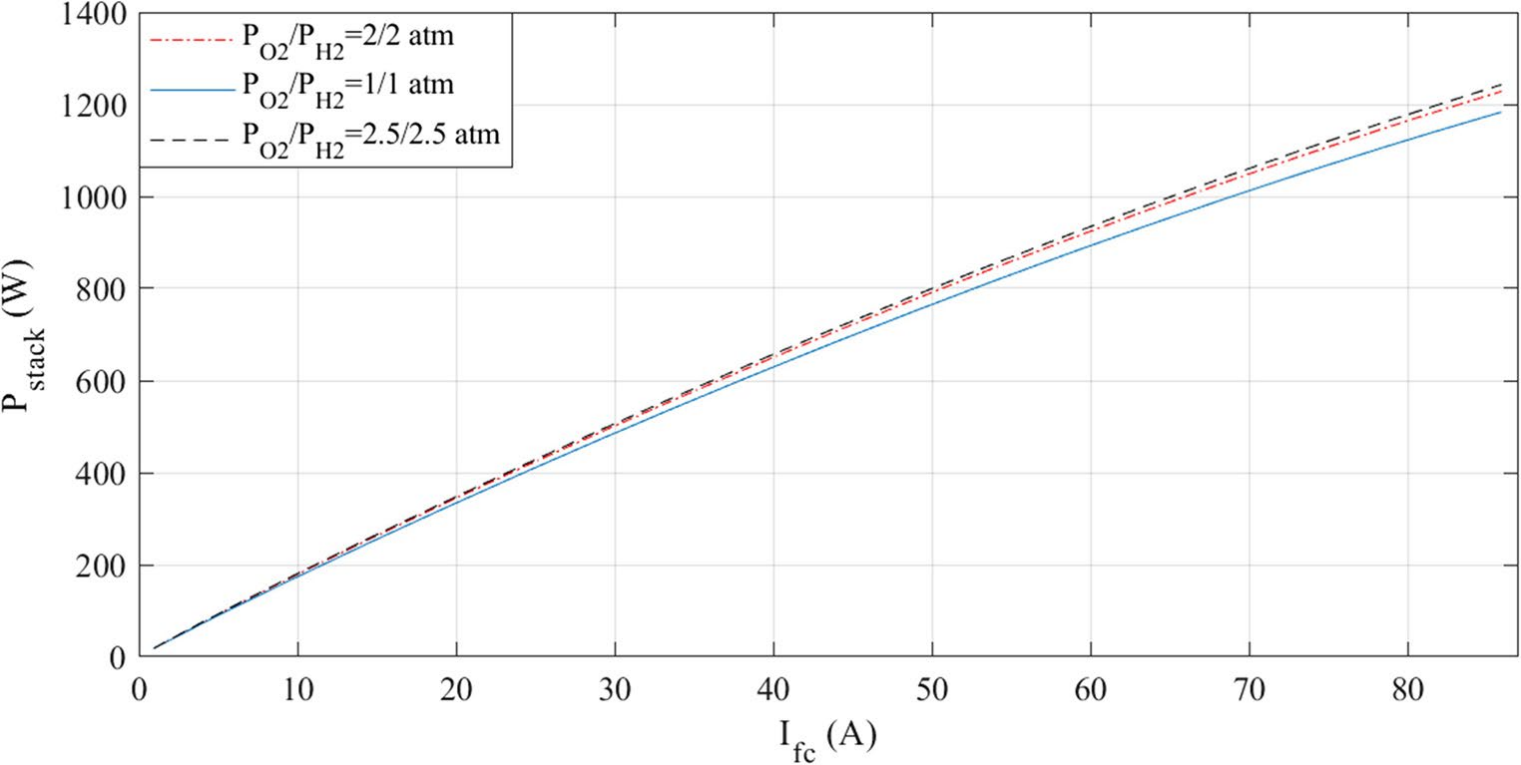
I-P curves at various pressures for Case 1.

The simulations are run at a constant temperature and with varying pressures. The I-V and I-P graphs are shown in Figs. 5 and 6. They visually represent the outcomes of these simulations. Analyzing these figures indicates a noticeable increase in voltage with pressure.

**Figure 7:**
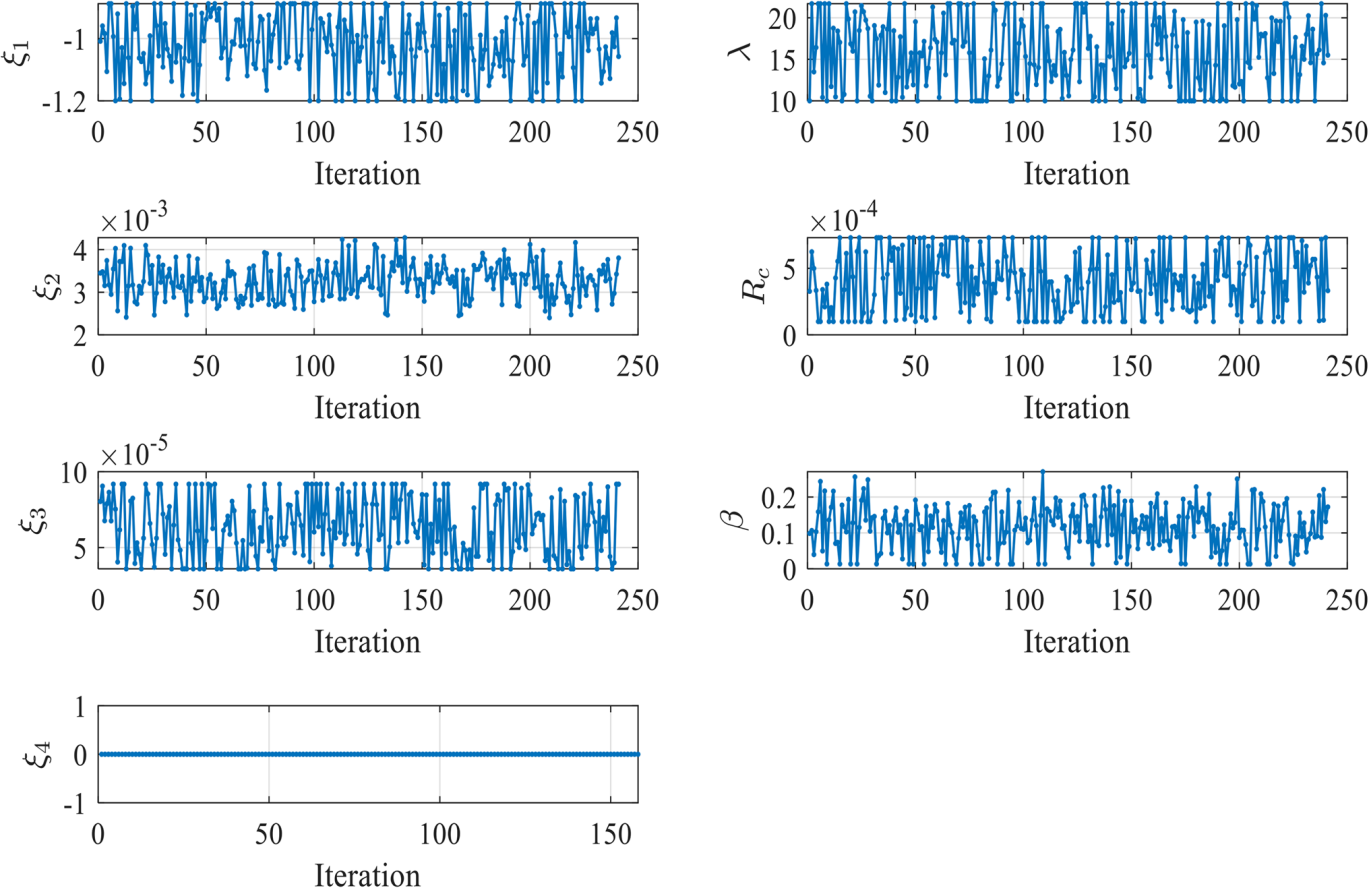
Values of Design Variables Used in Testing in Case 1.

Figure 7 illustrates the convergence of the design variables over 250 iterations in case 1. The seven design variables are denoted by zeta 0 – zeta 6 in Fig. 7. Each line represents a different design variable value, and the plot shows how these values stabilize as the simulation progresses. Ideally, the curves should remain relatively stable if the agent has found an effective parameter set during training. Fluctuations could suggest that the agent is still exploring slightly during testing or that the environment introduces variability that the agent must respond to by adjusting the parameters. The number of iterations (from 0 to 250) during testing is displayed on the X-axis (Iterations). The smallest SSE was achieved in the 60th iteration.

In contrast, the values of the seven 𝜁 parameters during these iterations are displayed on the Y-axis. The majority of the 𝜁 parameters (Zeta 0, 1, 2, 3, 5, and 6) exhibit stability with minimal variation during testing. This suggests that the PPO agent has effectively learned the optimal values for these parameters during training, and they remain relatively constant in the testing phase. Zeta 4 is the exception, showing significant fluctuations across the testing iterations. This could indicate that this parameter is more challenging for the agent to optimize or that it is highly sensitive to changes in the environment for the three tested cells.

**Figure 8:**
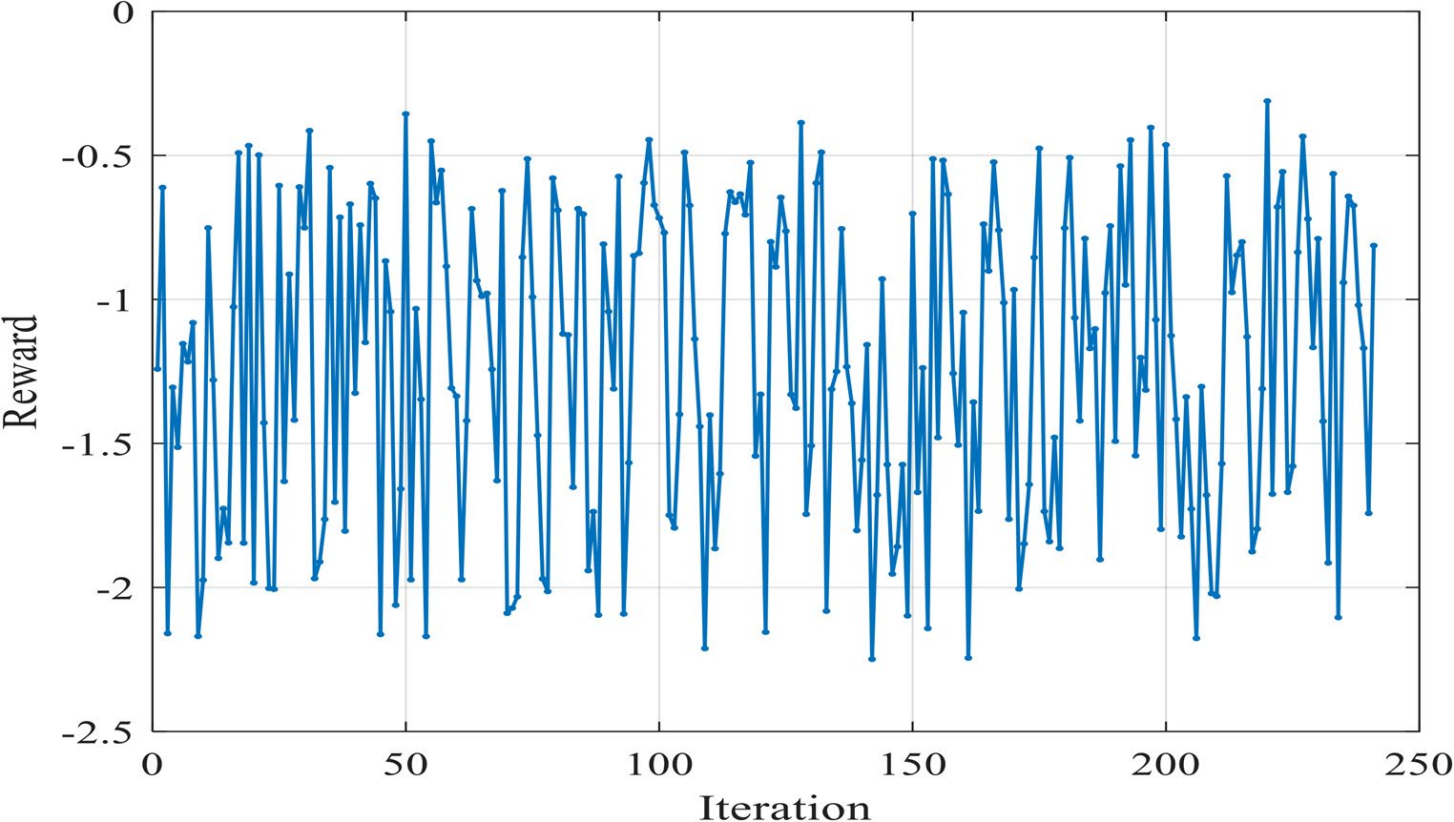
Reward Function Variation during Testing in Case 1.

Figure 8 illustrates the variation of the reward function during the testing in Case 1. The x-axis represents the iterations, while the y-axis shows the reward value. The testing reward curve reflects the agent’s ability to generalize its learned parameters to new situations.

**Figure 9:**
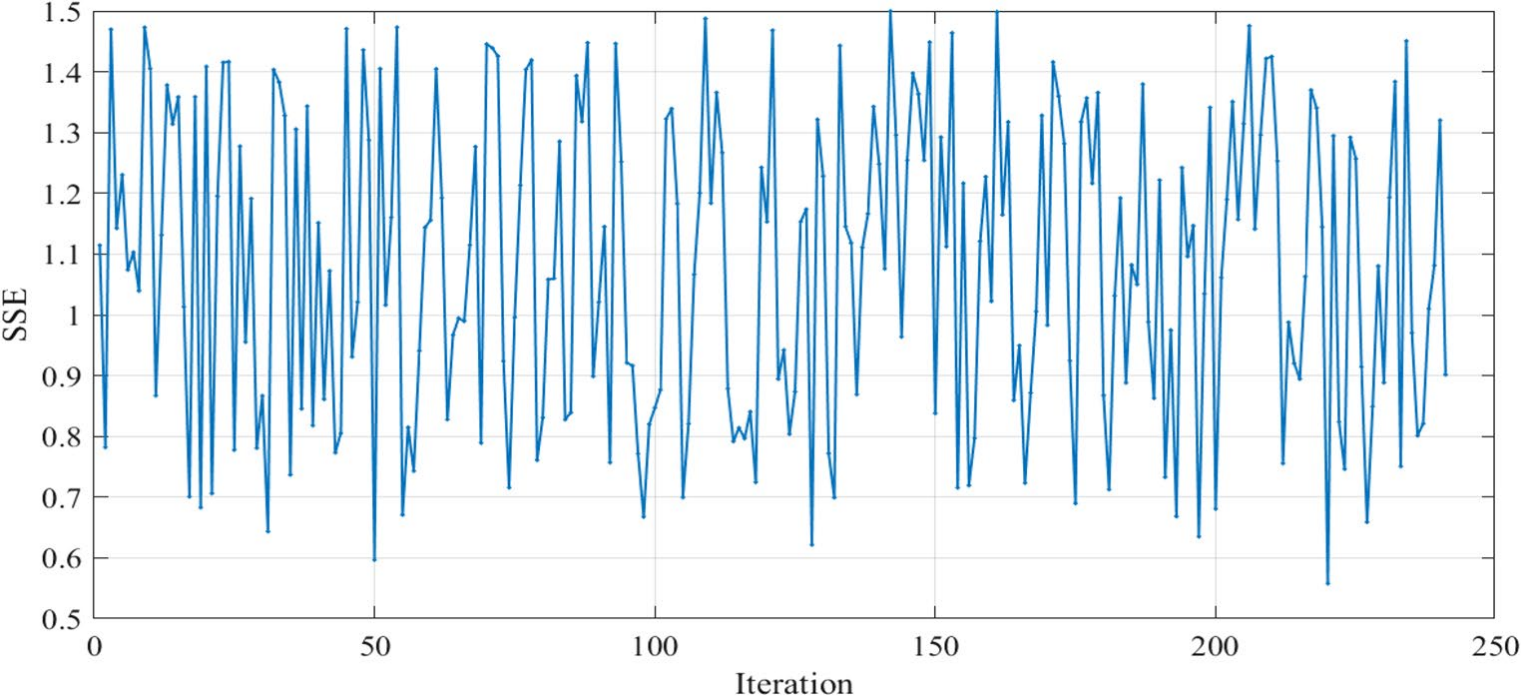
SSE computed through Testing in Case 1.

The SSE curves, Figs. 9 and 18, and Fig. 27, appear to fluctuate within a certain range dependent on each cell tested, indicating that the model is making predictions with varying levels of accuracy throughout the iterations to reach the optimized parameters with the lowest SSE value for each cell. The reward curve will similarly fluctuate as it is often closely tied to the SSE. Fluctuations during testing are a normal part of reinforcement learning, particularly in complex environments with some degree of stochasticity or variability, as in our case.

### Case study 2: 6 kW Nedstack PS6 PEMFC

The Nedstack PS6 has a power output of 6 kW, a membrane thickness of 1.78 mm, and 65 cells connected in series. The active area is 240 cm², with the highest current density being five A/cm². The best values obtained by the RL are compared to those acquired by the algorithms in Table 2. The SSE for the RL findings is lower.

**Table 2:** Design variables for case 2.

Parameter	RL	NNA^48^	SSO^48^	TSO^47^
$\:{\xi\:}_{1}$	-0.89999998	-0.8535	-0.9719	-0.8532
$\:{\xi\:}_{2}$	0.0028	2.4316	3.3487	2.461745
$\:{\xi\:}_{3}$	0.000054	3.7545	7.9111	3.94
$\:{\xi\:}_{4}$	-9.54E-05	-9.54	-9.5435	-9.54
λ	13.015454	13.0802	13	14.1357
$\:{R}_{c}$	1E-04	0.1	0.1	0.109423
β	0.0136	0.0136	0.0534	0.1139157
SSE	1.955545929	2.14487	2.18067	2.219

**Figure 10:**
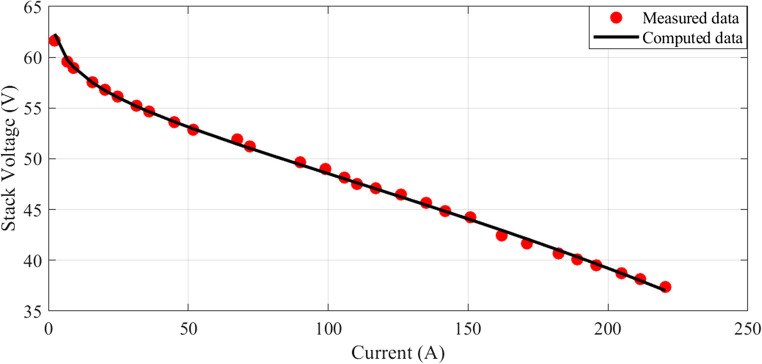
I-V Curves for Case 2.

**Figure 11:**
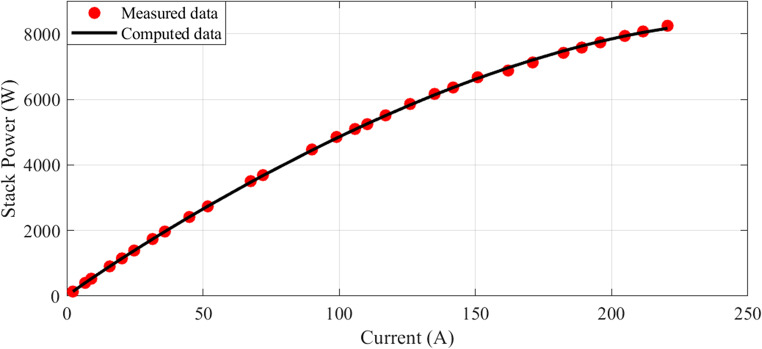
I-P Curves for Case 2.

The I-V characteristics are illustrated in Fig. 10, comparing estimated and measured values. A strong correlation between the calculated and measured values indicates a good match between the observed values and the model predictions. Similarly, the I-P curves are depicted in Fig. 11, with the calculated curve closely aligning with the observed data points, demonstrating a strong connection between the estimated values and the actual measurements.

**Figure 12:**
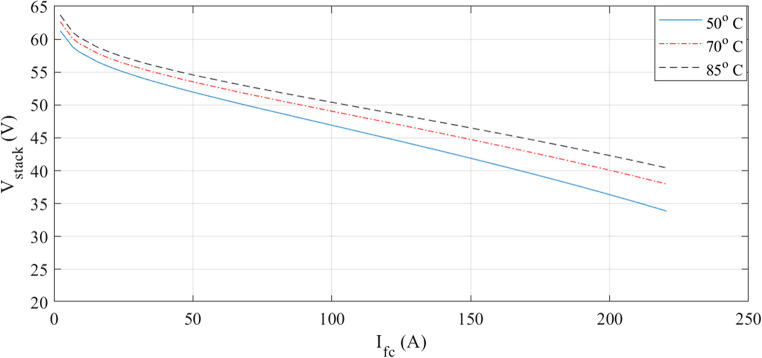
I-V curves at various temperatures for Case 2.

**Figure 13:**
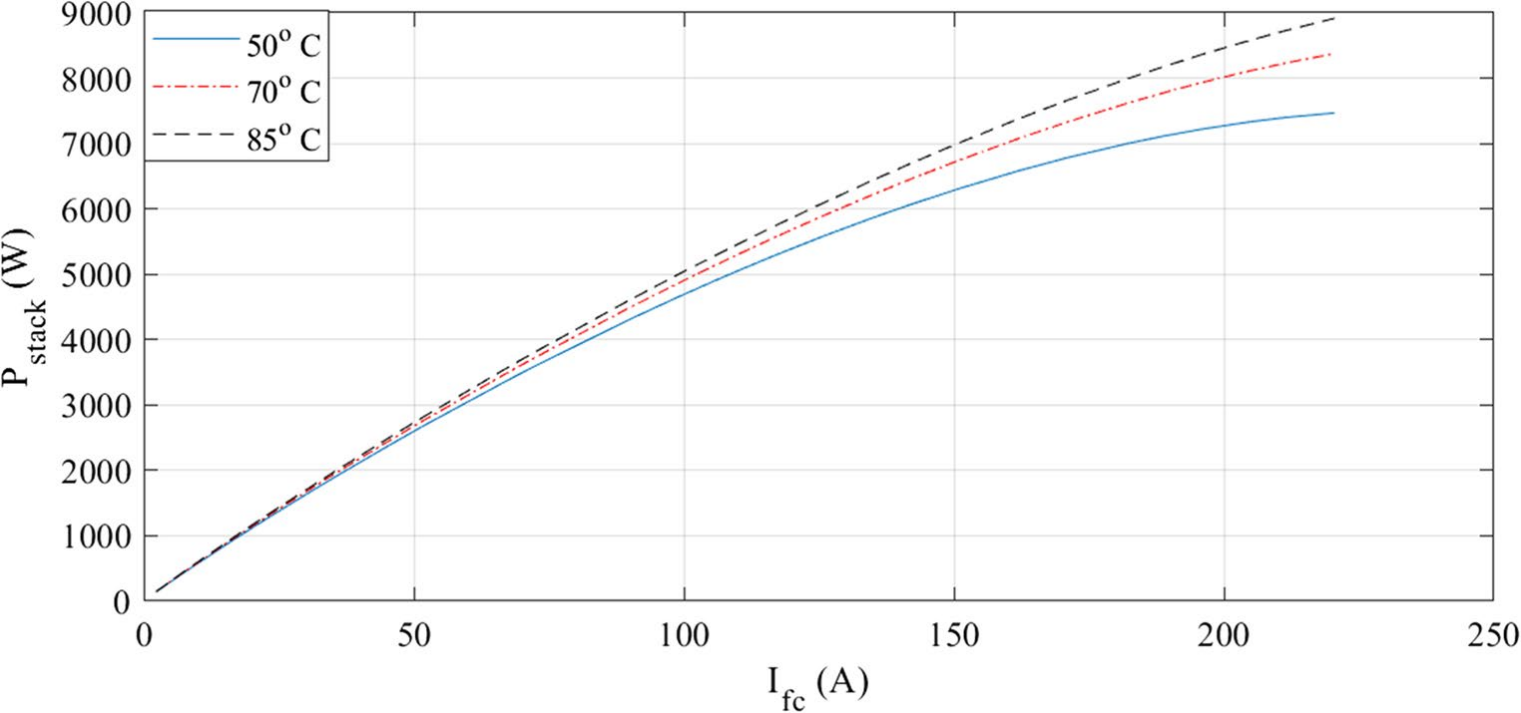
I-P curves at various temperatures for Case 2.

The characteristics at different temperatures are examined in the next two figures. An I-V curve comparison at 50, 70, and 85 °C is shown in Fig. 12. The voltage increases with temperature, as the curves demonstrate. Additionally, the comparison of I-P curves is shown in Fig. 13. As the temperature varies, so does the output power.

**Figure 14:**
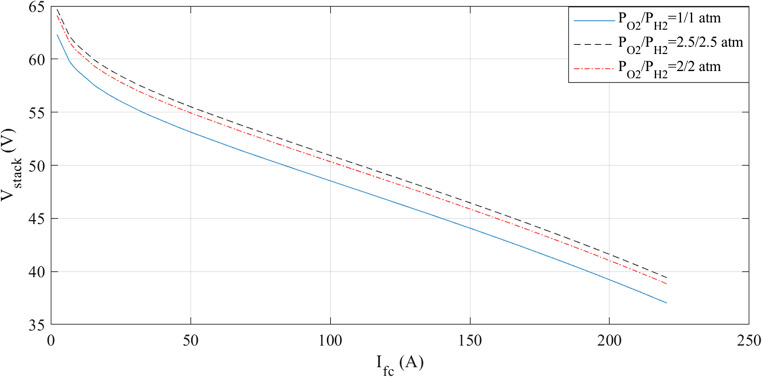
I-V curves at various pressures for Case 2.

**Figure 15:**
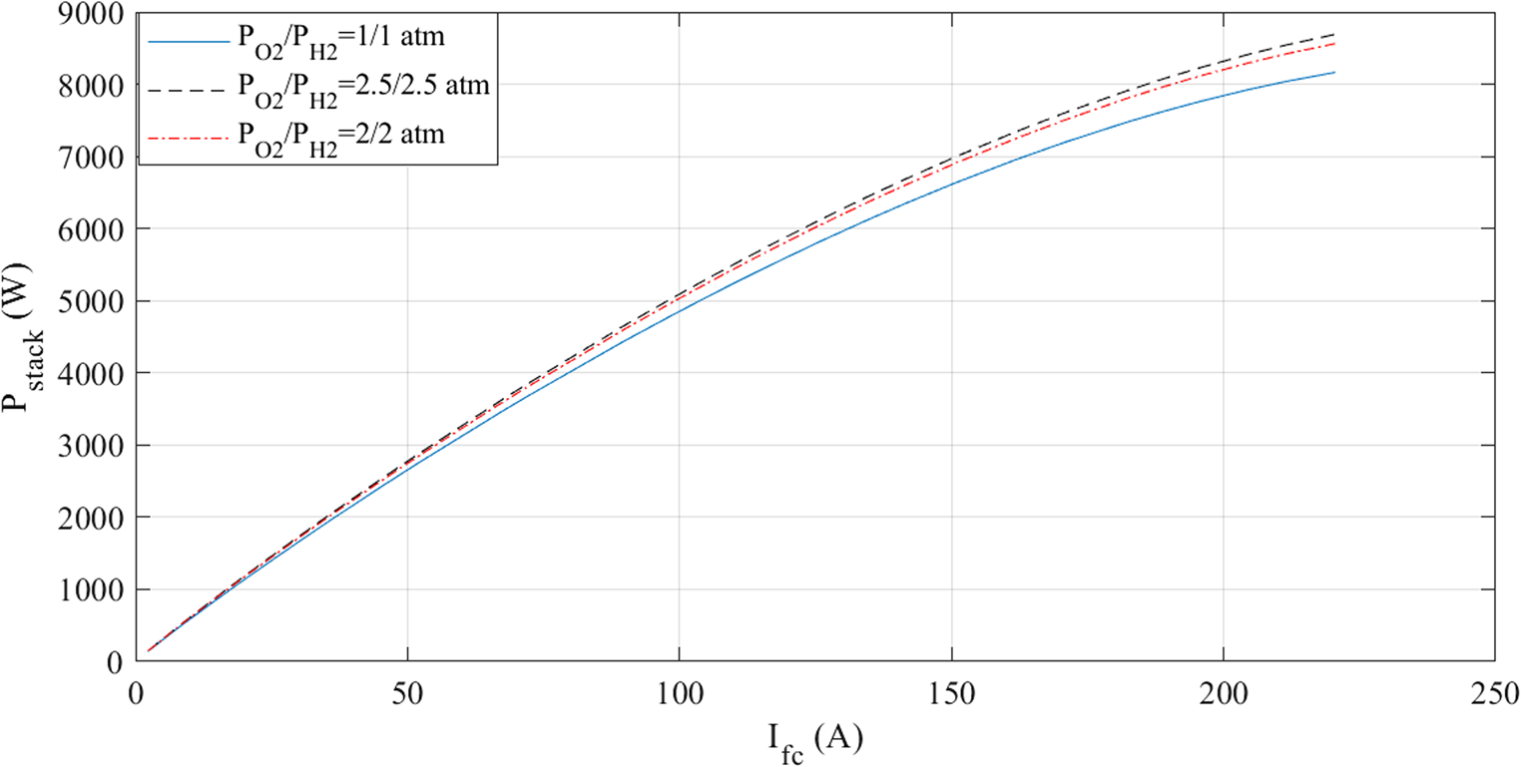
I-P curves at various pressures for Case 2.

The simulations are run at different pressures while keeping the temperature constant. Figures 14 and 15 present a graphic illustration of the results. An increase in voltage accompanies a rise in pressure.

**Figure 16:**
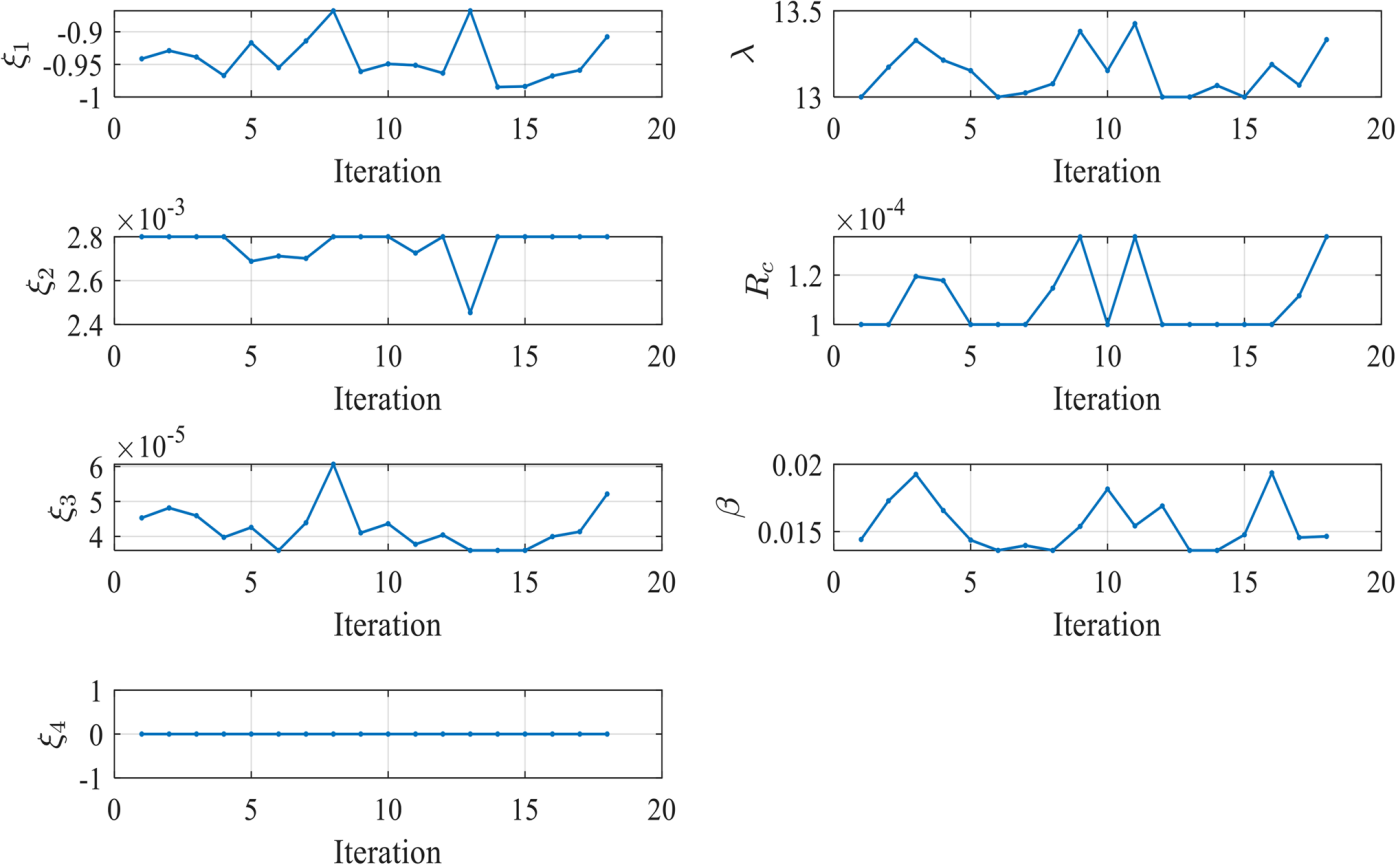
Values of Design Variables Used in Testing in Case 2.

**Figure 17:**
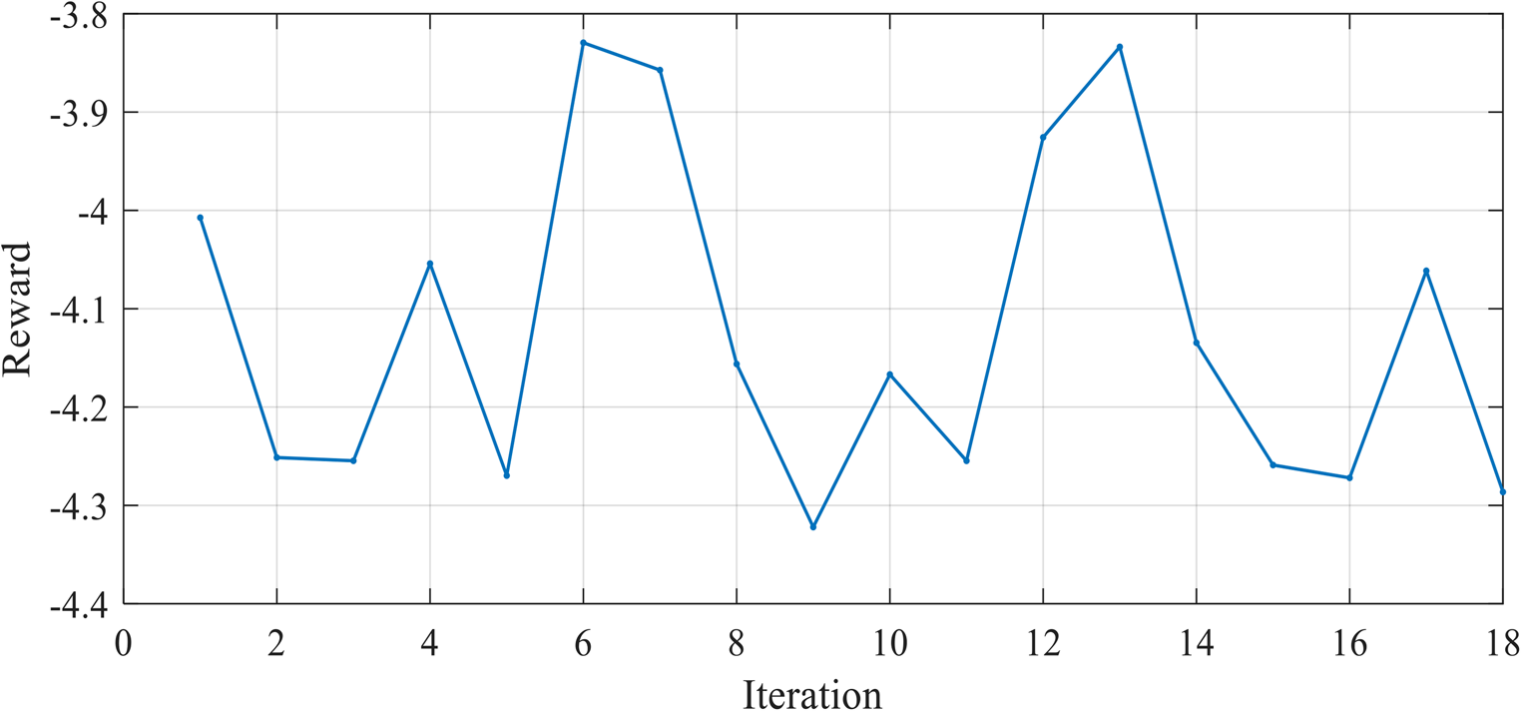
Reward Function Variation during Testing in Case 2.

**Figure 18:**
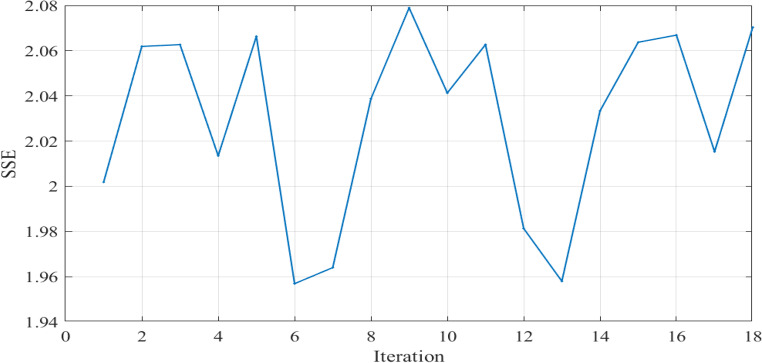
SSE computed through Testing in Case 2.

Figure 16 illustrates the convergence of the design variables over 17 iterations in case 2. Figure 17 illustrates the variation of the reward function during the testing in Case 2. The x-axis represents the iterations, while the y-axis shows the reward value. The smallest SSE was achieved in the 5th iteration as shown in Fig. 18. The seven design variables are denoted by zeta 0—zeta 6 in Fig. 16. Each line represents a different design variable value, and the plot shows how these values stabilize as the simulation progresses.

Figure 17 illustrates the variation of the reward function during the testing in Case 2. The x-axis represents the iterations, while the y-axis shows the reward value.

### Case study 3: Horizon H-12, 12 W PEMFC

The Horizon H-12 is a 12 W stack with 13 cells arranged in series and a 25 μm membrane thickness^48^. It has a maximum current density of 0.86 A/cm² and an active area of 8.1 cm². The best values obtained by the RL are listed in Table 3 and compared with those obtained by the competing algorithms. A lower SSE in the RL findings indicates more precise optimization.

**Table 3:** Design variables for case 3.

Parameter	RL	PSO^47^	TSO^47^	WOA^47^
$\:{\xi\:}_{1}$	-0.88785005	-1.0347536	-0.8532	-1.187
$\:{\xi\:}_{2}$	0.001859869	2.5449	1.571852	2.6697
$\:{\xi\:}_{3}$	4.87516E-05	6.32	3.61	3.6
$\:{\xi\:}_{4}$	-9.54E-05	-9.54	-9.54	-9.54
λ	14.682545	23	13.0243709	13.824
$\:{R}_{c}$	0.000173404	0.8	0.327874	0.8
β	0.17679477	0.1827039	0.17527388	0.1598
SSE	0.096572414	0.09658	0.09685	0.116

**Figure 19:**
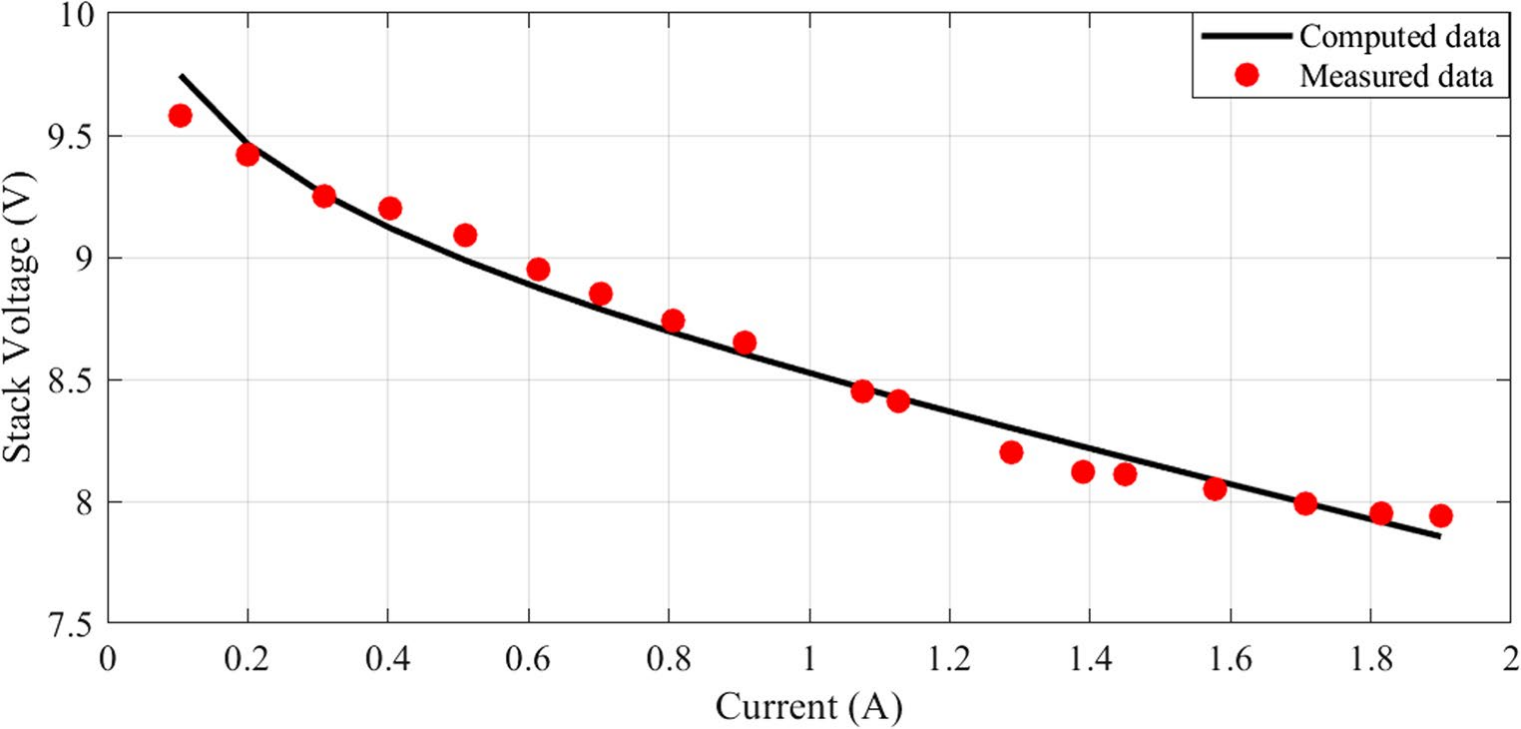
I-V Curves for Case 3.

**Figure 20:**
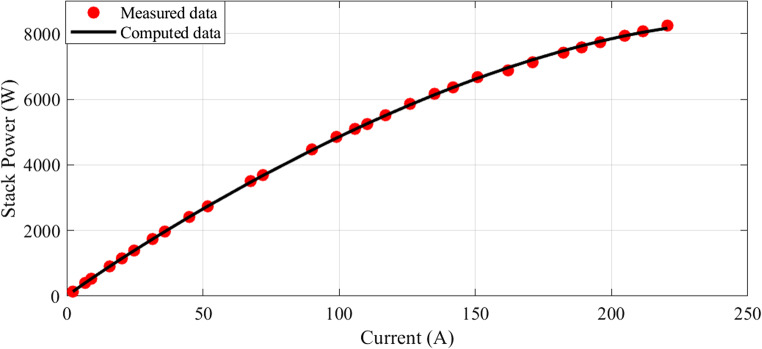
I-P Curves for Case 3.

The I-V characteristics are shown in Fig. 19, where observed values are compared with estimated values obtained from the model. The observed and calculated values show a high connection. Similarly, the I-P curves for the same PEMFC are demonstrated in Fig. 20. It indicates that there is a significant relationship between the estimated values and the actual measurements.

**Figure 21:**
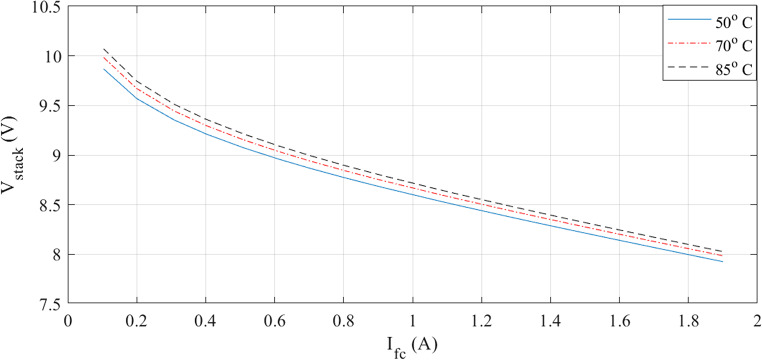
I-V curves at various temperatures for Case 3.

**Figure 22:**
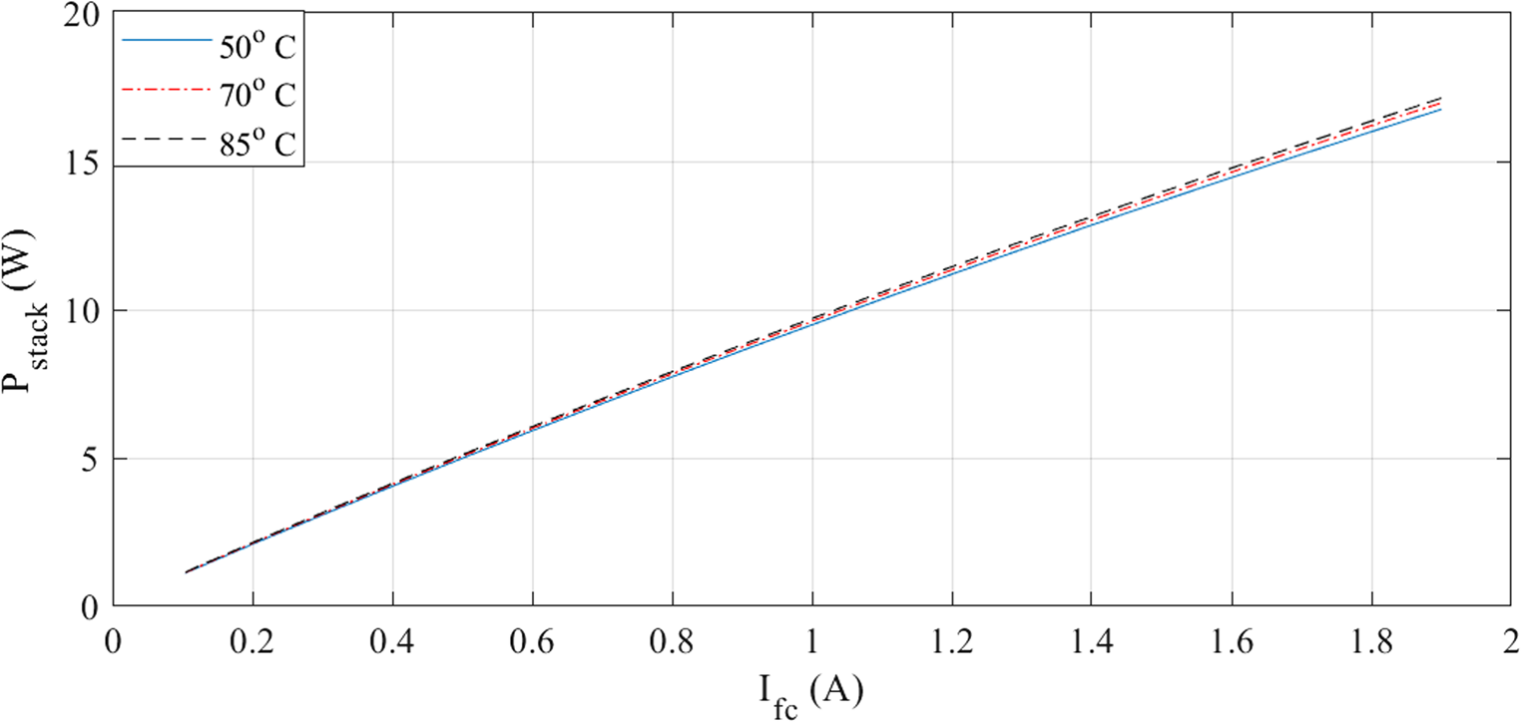
I-P curves at various temperatures for Case 3.

The properties at different temperatures are examined in the next two figures. I-V curves at temperatures of 50, 70, and 85° C are compared in Fig. 21. The voltage increases with temperature, as the curves demonstrate. Additionally, the comparison of I-P curves is shown in Fig. 22. A slight shift in output power is observed with a rise in temperature.

**Figure 23:**
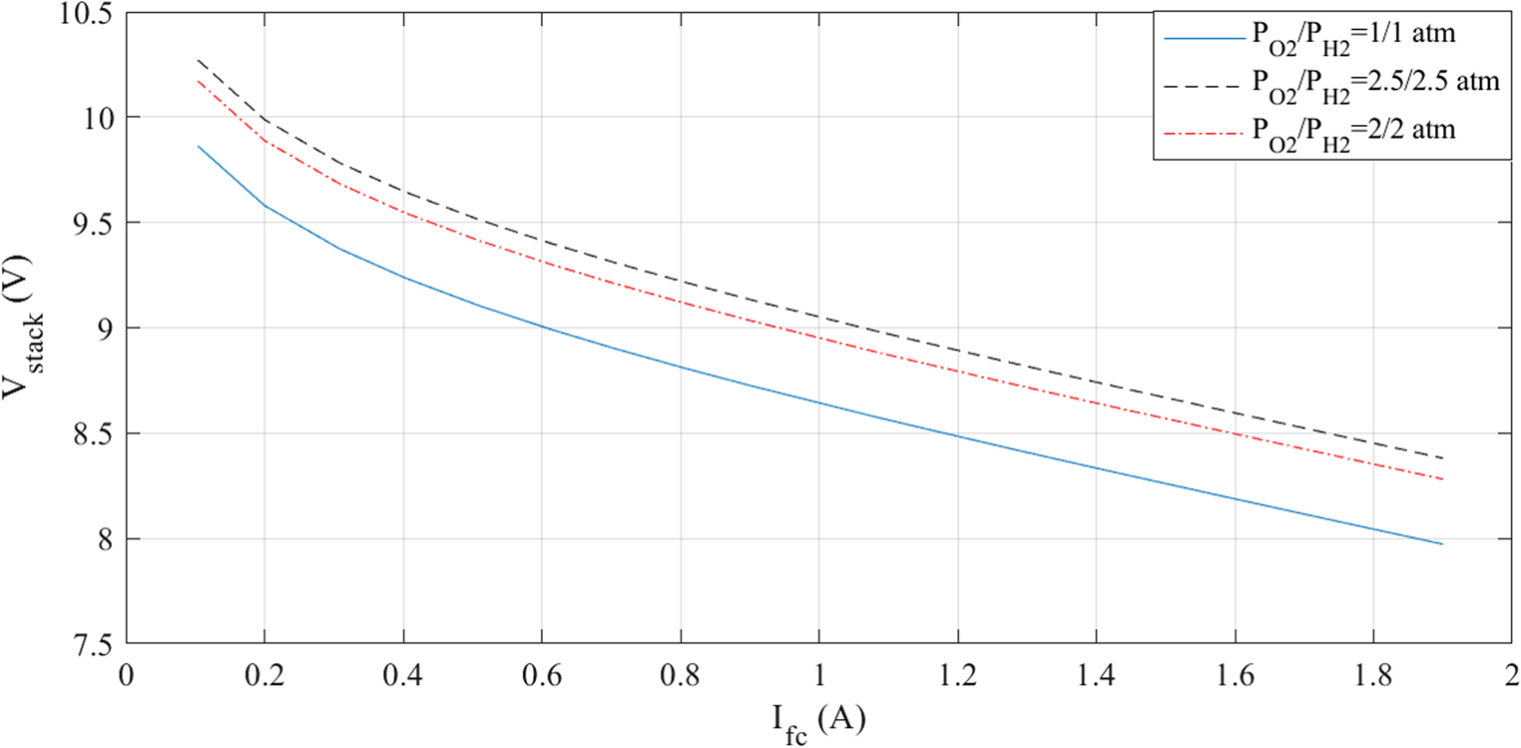
I-V curves at various pressures for Case 3.

**Figure 24:**
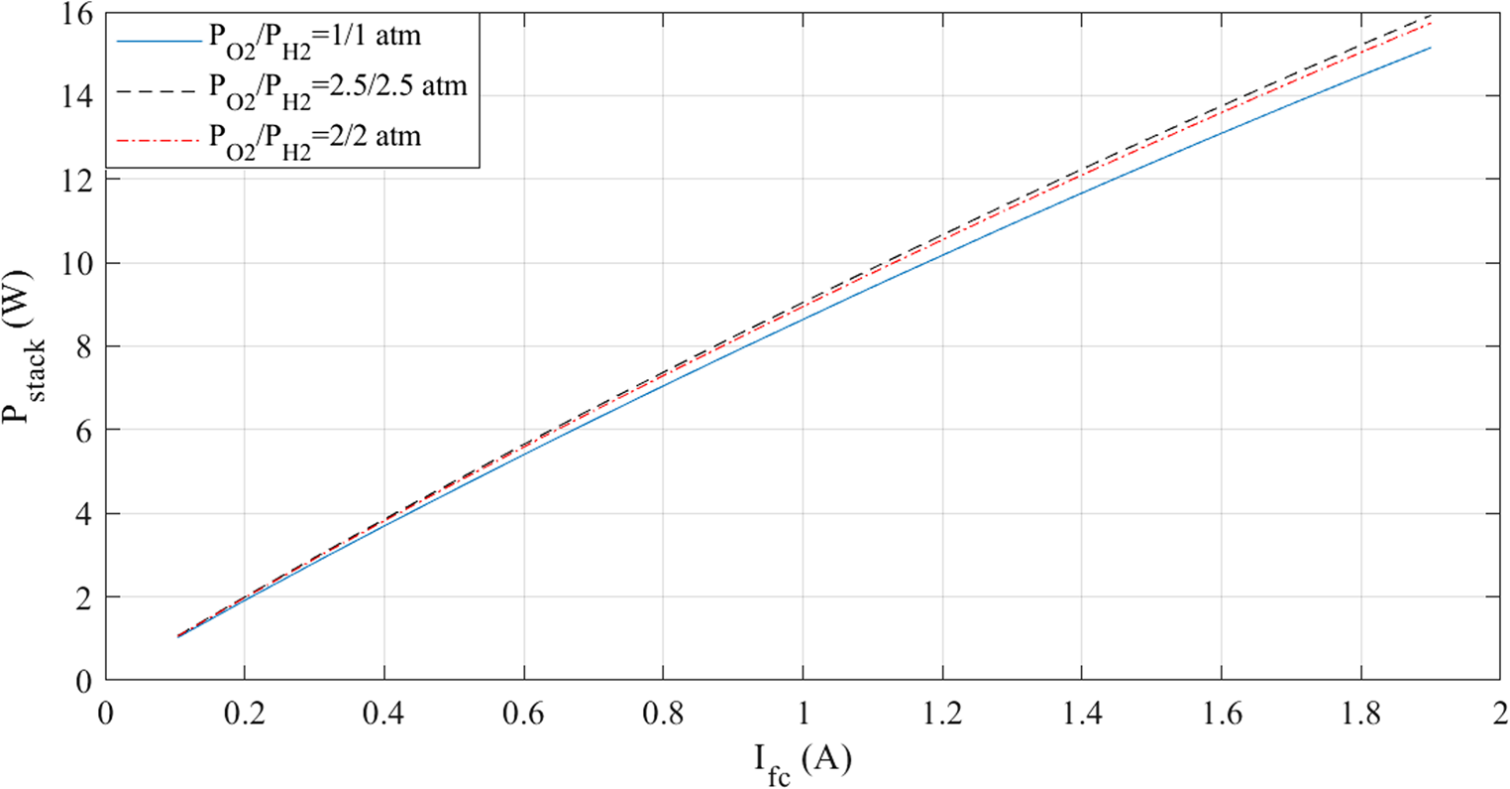
I-P curves at various pressures for Case 3.

At a fixed temperature, the simulations are run at different pressures. Figures 23 and 24 provide illustrations of the findings. An increase in voltage accompanies a rise in pressure.

**Figure 25:**
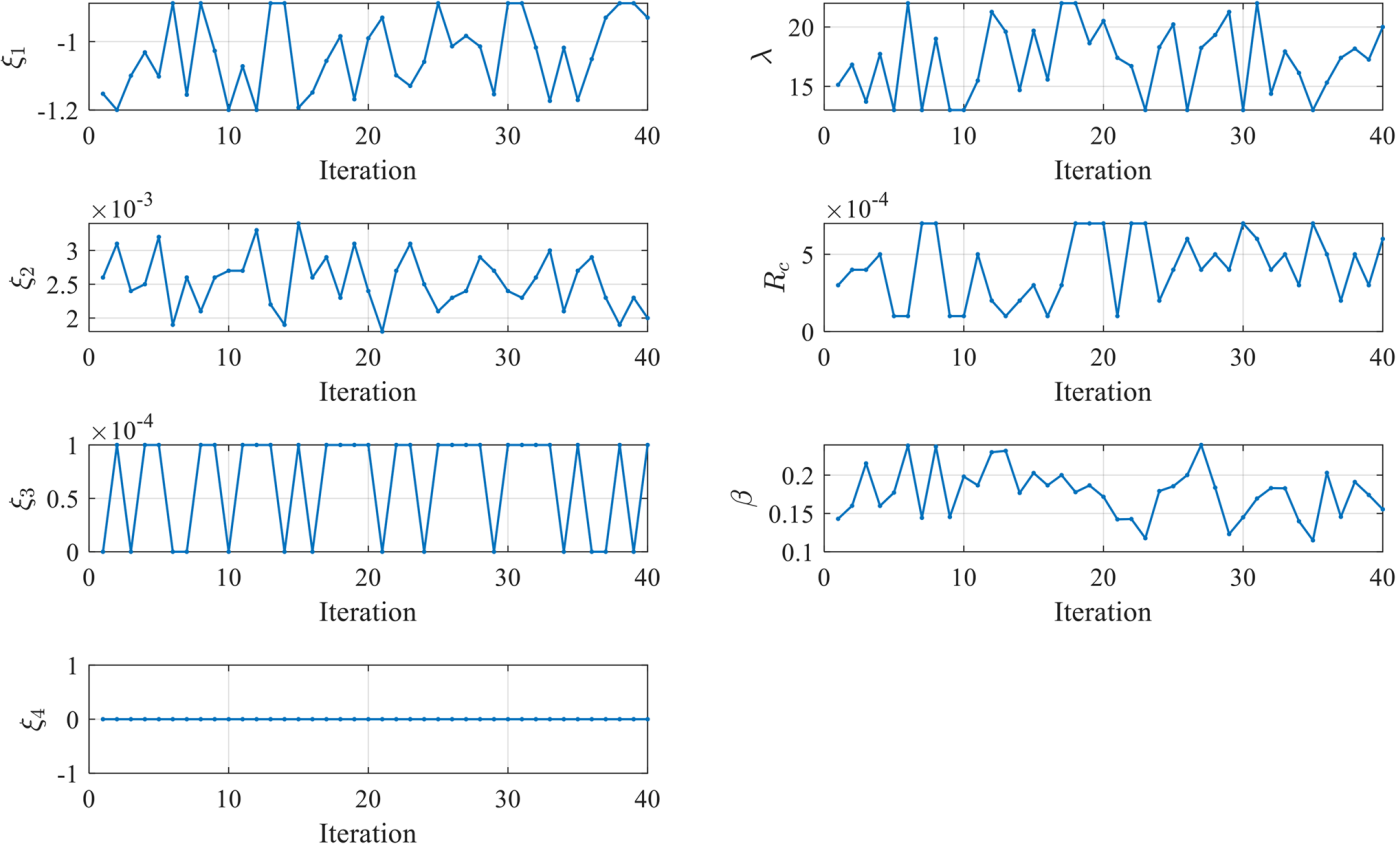
Values of Design Variables Used in Testing in Case 3.

**Figure 26:**
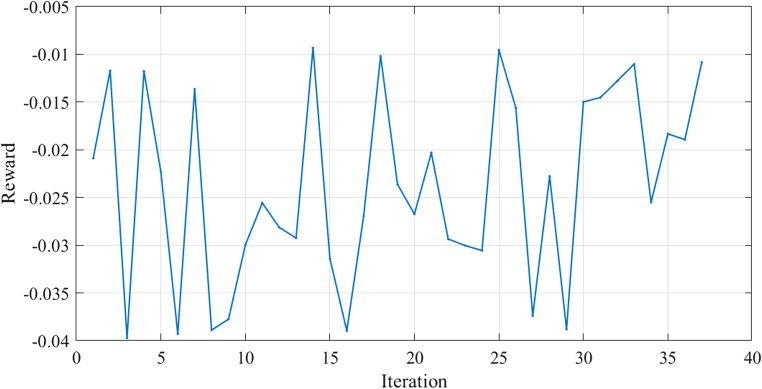
Reward Function Variation during Testing in Case 3.

**Figure 27:**
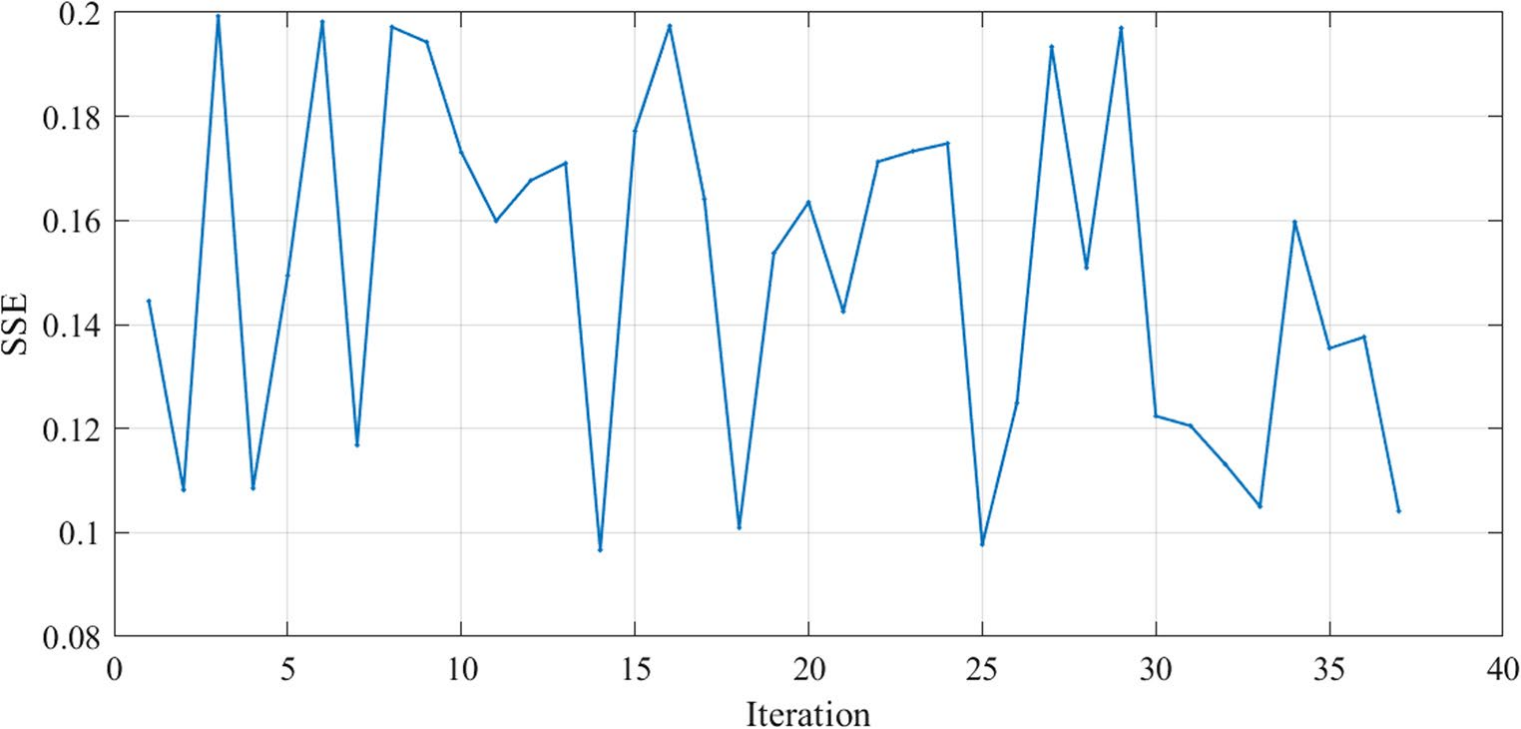
SSE Variation during Testing in Case 3.

Figure 25 illustrates the convergence of the design variables over 40 iterations in case 3. Figure 26 illustrates the variation of the reward function during the testing in Case 3. The x-axis represents the iterations, while the y-axis shows the reward value. The smallest SSE was achieved in 14th iteration as shown in Fig. 27. The seven design variables are denoted by zeta 0 – zeta 6 in Fig. 25. Each line represents a different design variable value, and the plot shows how these values stabilize as the simulation progresses.

Figure 26 illustrates the variation of the reward function during the testing in Case 3. The x-axis represents the iterations, while the y-axis shows the reward value.

### Statistical analysis

To further assess the performance of the RL-based parameter estimation approach for each PEMFC type, a comprehensive statistical analysis was conducted. The following metrics were employed:

- MSE: Measures the average squared difference between the estimated and measured voltages.
- MAE: Measures the average absolute difference between the estimated and measured voltages.
- RMSE: The square root of the MSE, providing another measure of the magnitude of errors.
- R-squared: Indicates the proportion of variance in the measured voltage explained by the estimated voltage.

The statistical metrics for the three PEMFC types are presented in Table 4.

**Table 4:** Statistical Metrics for the three PEMFC types.

	Case 1	Case 2	Case 3
Mean Squared Error (MSE)	0.0375	0.0674	0.005365
Mean Absolute Error (MAE)	0.122	0.201	0.0608
Root Mean Squared Error (RMSE)	0.194	0.26	0.0732
R-squared	0.998	0.999	0.981

The statistical analysis indicates that the RL-based approach effectively estimated parameters for all three PEMFC types. The R-squared values for all cases were high. This indicates that the estimated voltage closely follows the measured voltage. The MSE, MAE, and RMSE values varied across the cell types.

Furthermore, to assess the variability in the performance of the RL-based approach across multiple independent runs, the SSE was calculated for each case. The results are summarized in Table 5. It demonstrates some performance variability. The standard deviations ranging from 0.0012 to 0.0183. The overall performance of the RL-based approach remains consistent across the different cases. The mean SSE values for all cases are relatively low. This indicates that the agent can achieve accurate parameter estimation. These findings highlight the potential of the RL-based approach for reliable parameter estimation in PEMFCs.

**Table 5:** Summary of SSE Statistics for Multiple Independent Runs.

Metric	Case 1	Case 2	Case 3
Best SSE	0.5583	1.9555	0.0965
Worst SSE	0.611	1.9591	0.1457
Mean SSE	0.5826	1.9574	0.1034
Standard Deviation of SSE	0.0161	0.0012	0.0183

## Conclusions

The proposed PPO-based reinforcement learning approach successfully optimized prediction strategies for three different PEMFC cells, achieving the goal of developing a theoretical model that closely matches measured data. This article presented a parameter estimation for a PEMFC model, verified under a range of pressure and temperature conditions. The accuracy of the model was evaluated against experimental data and tested on commercial PEMFCs, including the Temasek 1 kW, the 6 kW Nedstack PS6, and the Horizon H-12 12 W. While the performance varied between cells, the agent was able to find optimal design variables for each, minimizing the SSE and improving voltage estimation. The proposed approach achieved an improvement in accuracy ranging from 3 to 48% in case 1, 10–23% in case 2, and up to 23% in case 3. To the knowledge of the authors, the use of reinforcement learning in PEMFC modeling has not been previously explored in the literature, making this study a novel contribution to the field. Fluctuations in the reward and SSE curves are expected due to the complexity and stochastic nature of the environment, but overall, the approach proved to be effective. Further work could focus on improving generalization across cells and refining the agent’s performance through targeted hyperparameter tuning and domain adaptation strategies.

## Supplementary Information

Below is the link to the electronic supplementary material.

Supplementary Material 1

## Data Availability

Dataset generated during the current study are available from the corresponding author on reasonable request.
