# Supplementary material for: Novel reinforcement learning technique based parameter estimation for proton exchange membrane fuel cell model
Source: Sci Rep. 2024 Nov 11;14:27475. doi: 10.1038/s41598-024-78001-5 (PMC11551159; doi:10.1038/s41598-024-78001-5)
Supplement: Supplementary file 1 — Supplementary Material 1 [file 41598_2024_78001_MOESM1_ESM.docx]

**Nomenclature:**

| Symbol/Abbreviation | Description |
| --- | --- |
| $\beta$ | Empirical coefficient for concentration overpotential |
| DG | Distributed Generation |
| $E_{Nernst}$ | Nernst potential |
| EWO | Enhanced Walrus Optimization |
| HHO | Harris Hawks Optimization |
| $I_{fc}$ | Fuel cell current |
| $J$ | Current density |
| KOA | Kepler Optimization Algorithm |
| $l$ | Membrane thickness |
| $M_{A}$ | Surface area |
| MPA | Marine Predator Algorithm |
| $N_{cells}$ | Number of series cells in the PEMFC stack |
| NNA | Neural Network Algorithm |
| $P_{H_{2}}$ | Partial pressure of hydrogen |
| $P_{O_{2}}$ | Partial pressure of oxygen |
| $R_{c}$ | Contact resistance |
| $R_{m}$ | Membrane resistance |
| RL | Reinforcement Learning |
| SSE | Sum of Squared Errors (objective function for optimization) |
| SSO | Social Spider Optimization |
| SOFC | Solid Oxide Fuel Cell |
| MAE | Mean Absolute Error |
| MCFC | Molten Carbonate Fuel Cell |
| MLP | Multi-Layer Perceptron |
| MSE | Mean Squared Error |
| PEMFC | Proton Exchange Membrane Fuel Cell |
| PPO | Proximal Policy Optimization |
| PSO | Particle Swarm Optimization |
| RMSE | Root Mean Squared Error |
| $T_{fc}$ | Temperature of the fuel cell |
| TSO | Transient Search Optimization |
| $V_{stack}$ | Voltage of the PEMFC stack |
| $v_{act}$ | Activation overpotential |
| $v_{conc}$ | Concentration overpotential |
| $v_{\Omega}$ | Ohmic loss (resistance-related voltage loss) |
| $\lambda$ | Membrane hydration level (design variable) |
| $\rho_{m}$ | Membrane resistivity |
| WOA | Whale Optimization Algorithm |
